# Nordic Walking Combined with Time-Restricted Eating Is Associated with Changes in Gut Microbiota Composition in Adults with Obesity—Pilot Study

**DOI:** 10.3390/nu18142373

**Published:** 2026-07-20

**Authors:** Alicja Nowak-Zaleska, Olga Czerwińska-Ledwig, Małgorzata Żychowska, Katarzyna Meyza, Dorota Łoboda, Tomasz Pałka, Agata Szlachetka, Ewa Ziemann, Tomasz Niewęgłowski, Anna Kurkiewicz-Piotrowska

**Affiliations:** 1 Department of Medical Sciences and Health, Kazimierz Wielki University, 85-091 Bydgoszcz, Poland; alicja.nowak-zaleska@ukw.edu.pl (A.N.-Z.);; 2 Institute for Basic Sciences, Faculty of Physiotherapy, University of Physical Culture, 31-571 Krakow, Poland; 3 Department of Genetics, Faculty of Biological Sciences, Kazimierz Wielki University, Powstańców Wielkopolskich 10, 85-090 Bydgoszcz, Poland; 4 Department of Physiology and Biochemistry, Faculty of Physical Education and Sport, University of Physical Culture, 31-571 Krakow, Poland; 5 Faculty of Medicine and Health Sciences, University of Applied Sciences in Tarnow, 33-100 Tarnów, Poland; 6 Department of Athletics, Strength and Conditioning, Poznań University of Physical Education, 61-871 Poznań, Poland; 7 ETER-MED, Non-Public Healthcare Centre, 80-822 Gdansk, Poland

**Keywords:** physical activity, Nordic walking, time-restricted eating, genetic taxonomy, intestinal microbiota, gut microbiome, obesity

## Abstract

Background/Objectives: Rearrangement of the gut microbiota toward a symbiotic profile may be influenced by physical activity and diet in both healthy and obese individuals. This study aimed to characterize gut microbiota using next-generation sequencing (NGS) and to evaluate the effect of a 6-week Nordic Walking (NW) program combined with Time-Restricted Eating (TRE; 10 h eating window) in individuals with obesity. Methods: The study included healthy controls (C; *n* = 10; 64.7 ± 6.7 years) and individuals with obesity (A; *n* = 10; 60.0 ± 4.5 years). The intervention consisted of three moderate-intensity NW sessions per week, individually adapted to participants’ capacity. Gut microbiota was analyzed using nanopore 16S rRNA sequencing (V3–V9 regions). Results: Baseline microbial composition differed significantly between obese and control groups, with Bray–Curtis dissimilarity ranging from 49.98% (phylum) to 65.97% (species) (*p* ≤ 0.02). After intervention, within-group dissimilarity in the obese cohort (A vs. B) decreased to 25.24–51.86% but was not significant (*p* = 0.88–1.00). Post-intervention comparisons (B vs. C) still showed significant differences at higher taxonomic levels, including class (44.10%, *p* = 0.015), order (31.62%, *p* = 0.022), family (50.48%, *p* = 0.011), genus (58.09%, *p* = 0.005), and species (63.47%, *p* = 0.0003). Alpha diversity showed no significant differences at species and genus levels, but significant group effects were observed at higher ranks, including order (Shannon *p* = 0.01; Simpson *p* = 0.01), class (Simpson *p* = 0.02), and phylum (Shannon *p* = 0.02). Conclusions: The NW + TRE intervention was associated with partial normalization of gut microbiota structure and reduced microbial dissimilarity, suggesting a shift toward a more symbiotic profile; however, differences compared with controls persisted, indicating incomplete convergence after 6 weeks.

## 1. Introduction

A review of the literature concerning the taxonomic composition of the human gut microbiota indicates the predominance of representatives of seven major phyla: *Firmicutes*, *Bacteroidetes*, *Actinobacteria*, *Fusobacteria*, *Proteobacteria*, *Verrucomicrobia*, and *Cyanobacteria*. Among these, *Firmicutes* and *Bacteroidetes* constitute the most abundant microbial populations within this environment (exceeding 90%) and are regarded as components of the symbiotic microbiota characteristic of healthy adults [1]. Studies have also demonstrated that microorganisms belonging to the phylum *Proteobacteria* represent the most unstable bacterial population within the intestinal ecosystem, and their representatives are considered markers of dysbiotic microbiota [2].

Since the advancement of sequencing technologies, numerous research groups have reported that the microbiota composition of diseased individuals undergoes substantial alterations, and that these changes constitute one of the major factors contributing to the pathogenesis of many disorders. This condition is referred to as dysbiosis or dysbacteriosis. It is defined as a state of imbalance between microbial populations constituting the natural human intestinal microbiota and factors affecting host health. Dysbiosis has been associated with numerous diseases, including obesity, diabetes mellitus, autoimmune disorders, and inflammatory bowel disease [3,4,5].

At present, microbiota composition can be investigated not only through culture-based methods but also through analyses based on the genetic material of microorganisms inhabiting various environments. Bacterial identification relies on sequencing specific fragments of microbial genomes [6,7]. The most commonly investigated sequence is the 16S rRNA gene, widely regarded as the “gold standard.” Supplementary Table S1 summarizes the methods, sequencing technologies, molecular markers, and reference databases used for taxonomic identification and microbiota analysis [6,7,8,9,10,11,12,13,14,15,16,17,18,19,20,21,22,23,24,25].

Evidence accumulated over the past years has identified the gut microbiome as an important factor associated with obesity; however, the extent of its contribution to obesity, particularly in the context of obesity-related comorbidities, remains uncertain [26,27].

Studies have demonstrated that individuals with obesity exhibit a gut microbiota profile distinct from that observed in individuals with normal body weight [28]. Meta-analyses evaluating the gut microbiota composition of obese and non-obese individuals indicate reduced microbial diversity and altered composition at both the phylum and genus levels in obesity. At the phylum level, adults with obesity generally demonstrate a higher abundance of *Firmicutes* and a lower abundance of *Bacteroidetes* compared with non-obese individuals. Although some studies have confirmed reduced diversity and compositional differences in the gut microbiota of both obese and non-obese adults, the substantial heterogeneity observed across studies limits the ability to draw unequivocal conclusions. Reported alterations include lower relative abundances of the genera *Bifidobacterium* and *Eggerthella*, as well as higher relative abundances of *Acidaminococcus*, *Anaerococcus*, *Catenibacterium*, *Dialister*, *Dorea*, *Escherichia-Shigella*, *Eubacterium*, *Fusobacterium*, *Megasphaera*, *Prevotella*, *Roseburia*, *Streptococcus*, and *Sutterella* [29].

Among the factors implicated in obesity, including environmental, lifestyle-related, genetic, and microbiome-associated determinants, dietary factors are also considered highly important [30,31]. According to Manoogian, both consistency of the daily eating window and the absence of prescribed caloric restriction constitute important determinants of dietary intervention efficacy [32]. Time-Restricted Eating (TRE) limits food intake to a defined daily eating window without requiring intentional restriction of either dietary composition or caloric intake [32,33]. In contrast, intermittent fasting (IF), in which adherence to a specific dietary regimen is considered essential, as reported by Paukkonen et al. [34] and Zeb et al. [35], may influence gut microbiota composition and consequently affect the numerous physiological processes in which the gut microbiota is involved.

A review of the literature concerning the role of physical activity in modulating gut microbiota composition toward a more symbiotic profile indicates a mutually beneficial relationship between exercise and the gut microbiota [27,36]. Importantly, this effect has been observed in training programs such as Nordic Walking (NW), which is considered safe for individuals across a broad spectrum of health conditions [37,38]. The combination of a Nordic Walking intervention with a 14:24 TRE dietary strategy resulted in favorable changes in body composition, particularly reductions in body weight and adipose tissue mass, with more pronounced effects observed after 12 weeks compared with 6 weeks of intervention. Concurrently, beneficial alterations in selected adipokines, including reduced resistin concentrations, were observed, potentially indicating improved metabolic regulation. As emphasized by the authors, the combined intervention model was well tolerated and did not induce adverse changes in basic health parameters [39].

Considering the global prevalence of obesity, the variety of proposed dietary strategies (including IF and TRE), and the feasibility of implementing structured exercise programs, we aimed to investigate whether a 6-week NW training program would induce favorable alterations in the gut microbiota of individuals with obesity. In addition, we sought to integrate two practical and easily implementable lifestyle interventions, Nordic walking training and time-restricted eating, in order to potentially enhance or accelerate the onset of beneficial gut microbiota adaptations, which in exercise-only interventions are typically reported after longer intervention periods of approximately 12 weeks [40,41]. Given currently available methods for microbiota analysis, we employed 16S rRNA gene sequencing using NGS technology for microbial identification. Based on the existing literature, we hypothesized that the gut microbiota of individuals with normal body weight and those with obesity would differ, and that microbial diversity would be lower in individuals with obesity compared with healthy controls. Furthermore, we hypothesized that the gut microbiota composition of individuals with obesity would differ qualitatively and quantitatively before and after completion of the 6-week NW training program combined with TRE. We also assumed that the quantitative composition of dysbiotic microbiota, as well as the *Firmicutes/Bacteroidetes* ratio, would change in a direction reflecting a shift in the microbiota profile of individuals with obesity toward that observed in healthy individuals. The findings of the present study may contribute to the development or optimization of obesity treatment strategies and preventive interventions.

## 2. Materials and Methods

### 2.1. Study Design

This study was designed as a prospective, longitudinal, quasi-experimental pre–post intervention trial with a non-randomized control group. Participants were assigned to an obesity group or a normal-weight control group according to body mass index (BMI). Both groups were assessed at baseline to compare clinical and microbiota-related parameters. Only the obesity group underwent the 6-week intervention, whereas the normal-weight group was evaluated only once and served as a cross-sectional reference. Post-intervention outcomes in the obesity group were compared with both baseline values (within-group analysis) and the baseline measurements of the normal-weight group (between-group analysis).

### 2.2. Characteristics of the Participants Group and Healthy Controls

Participants were considered eligible for enrollment if they met the predefined inclusion criteria. For the obesity group, eligibility required a body mass index (BMI) greater than 30.0 kg/m^2^, whereas participants assigned to the control group were required to present a BMI within the normal range (18.5–24.9 kg/m^2^). Additional inclusion criteria included the absence of contraindications to either structured physical exercise or time-restricted eating interventions, as well as voluntary consent to participate in the study. Inclusion and exclusion criteria are listed in Table 1.

**Figure 1:**
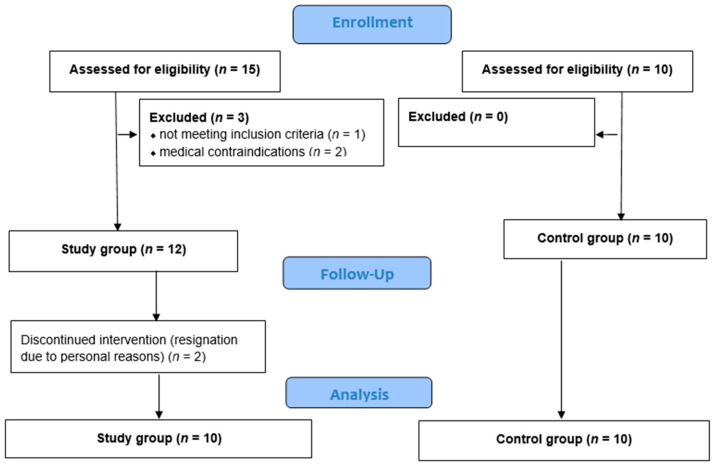
Flow diagram of participant recruitment, allocation, follow-up, and analysis in the study.

Prior to enrollment, all candidates underwent medical screening aimed at identifying potential contraindications to participation in the intervention protocol. Ultimately, 20 participants fulfilling all eligibility criteria were enrolled in the study (Figure 1). Baseline demographic and clinical characteristics of the study population are presented in Table 2.

All study procedures were conducted in accordance with the principles of the Declaration of Helsinki and applicable national regulations governing research involving human participants. The study protocol was approved by the Bioethics Committee (No. 59/KBL/OIL/2024, approved on 4 February 2024). Prior to enrollment, all participants received detailed verbal and written information regarding the study objectives, procedures, potential risks, and anticipated benefits. Written informed consent was obtained from all participants before study participation. Participants were also informed of their right to withdraw from the study at any time without providing a reason and without any consequences regarding their access to standard medical care.

### 2.3. Training Protocol

The Nordic walking (NW) training program was conducted outdoors during the spring and summer seasons by a qualified instructor, in accordance with the protocol described in our previous publications [38,39]. Exercise intensity was maintained at 60–70% of maximal heart rate (HR_max_), which corresponds to moderate intensity, individually calculated using the formula proposed by Nes et al. (2013) [42]. Heart rate was continuously monitored using sports testers (M400, Polar, Kempele, Finland) programmed with individual participant data, including the target heart rate range. Whenever heart rate exceeded the predefined limits, the device emitted an audible signal, prompting the instructor to adjust the exercise load to maintain moderate-intensity effort within the prescribed range.

The six-week structured health-training program comprised 18 sessions performed three times weekly in the morning hours, with each session lasting approximately 60 min. Beginning with the fourth session, both walking duration and distance were progressively increased while maintaining proper Nordic walking technique. Each training session consisted of a 10 min warm-up including low-intensity general and stretching exercises (Borg scale: 6–8), followed by the main exercise phase lasting up to 45 min at moderate intensity (60–70% HRmax; Borg scale: 10–12). During this phase, participants practiced and reinforced proper Nordic walking technique while gradually extending walking duration within the prescribed heart rate range. Each session concluded with a 5 min cool-down period (Borg scale: 6–8) consisting of stretching exercises to facilitate recovery. Participants were included in the final analysis only if they attended a minimum of 17 out of 18 training sessions.

### 2.4. Time-Restricted Eating

Participants were instructed to follow a daily 14 h fasting period, scheduled according to individual preference, with all meals consumed within a 10 h eating window. Particular attention was paid to ensuring that dietary modification was limited solely to the eating window, without introducing any additional interventions, so as not to influence the study outcomes in order to avoid Hawthorne effect [43]. No additional dietary modifications were introduced, and participants were asked to maintain their habitual dietary patterns throughout the intervention period. Adherence to the time-restricted eating (TRE) regimen was supervised by the instructor during each training session. Participants were asked whether they were adhering to the protocol and were given the opportunity to clarify any concerns or difficulties they encountered.

### 2.5. Sample Collection and Preparation

Stool samples were collected at baseline, immediately prior to initiation of the training intervention (groups A and C), and again following completion of the intervention period in the obesity group (group B). Participants received sterile plastic collection containers for sample acquisition. Following collection, all specimens were stored at −80 °C until DNA isolation.

DNA isolation from fecal samples was performed using the Fecal DNA Extraction Kit (IBI Scientific, Dubuque, IA, USA) according to the manufacturer’s instructions, with minor protocol modifications introduced to improve cell lysis efficiency and removal of PCR inhibitors [38]. Specifically, an additional mechanical and thermal pre-treatment step was applied, in which stool samples were suspended in ST1 buffer, vortexed, and incubated at elevated temperature (70 °C), followed by extended high-speed vortexing in a horizontal position to enhance microbial cell disruption. Furthermore, an additional centrifugation step was introduced after initial clarification to improve phase separation and reduce residual particulate material.

In addition, an extended inhibitor removal procedure was implemented by increasing incubation time at low temperature (0–4 °C) after addition of ST2 buffer, which improved precipitation of inhibitory compounds commonly present in fecal material. The purification workflow was further modified by incorporating an additional washing step with ST3 buffer applied directly to the GD column prior to final washing steps with Wash Buffer, thereby increasing DNA purity. Finally, the elution step was optimized by extending incubation time before final centrifugation to enhance DNA yield [38]. Extracted DNA samples were subsequently stored at −20 °C pending further molecular analyses.

### 2.6. Sequencing and Bioinformatics

The V3–V9 hypervariable regions of the bacterial 16S rRNA gene were amplified using the universal primer pair 337F/1391R. Amplicons were barcoded using the Oxford Nanopore Native Barcoding Expansion Kit and sequencing libraries were prepared according to the Oxford Nanopore ligation protocol. Sequencing was performed by GeneXone S.A. (Poznań, Poland) on MinION R9.4.1 flow cells (Oxford Nanopore Technologies, Oxford, United Kingdom) using validated in-house procedures.

Taxonomic assignment of sequencing reads was carried out using the UBLAST algorithm based on comparisons with reference sequences from the NCBI nucleotide database. Reads that could not be assigned unambiguously at the species level were classified to the closest matching taxonomic rank supported by the reference database (e.g., genus or family) or excluded from downstream analyses if no reliable assignment was obtained.

Taxonomic profiles were exported as a classification table compatible with the Pavian interactive visualization platform [44]. Microbial diversity was evaluated using the Simpson diversity index across multiple taxa read-count thresholds (0, 5, 15, 30, and 50 reads) using different minimum read-count thresholds to assess the influence of rare taxa on diversity estimates. Hierarchical clustering analysis was performed using the ClustVis platform [45].

### 2.7. Statistical Analysis

Baseline characteristics of the study participants were compared using the independent-samples Student’s *t*-test, with effect size additionally assessed using Cohen’s d.

Both alpha and beta diversity were evaluated in the statistical analysis. Alpha diversity was assessed using the Shannon and Simpson (1-D) diversity indices, whereas beta diversity was determined based on the Bray–Curtis dissimilarity metric. The pre–post comparison within the obesity group (A vs. B) was based on repeated measurements obtained from the same participants. Therefore, paired analyses of alpha-diversity indices were performed using the Wilcoxon signed-rank test. Comparisons between independent groups (A vs. C and B vs. C) were performed using the Mann–Whitney U test. Beta-diversity analyses were performed using Bray–Curtis distance matrices and analyzed with ANOSIM and PERMANOVA (NPMANOVA) to assess overall differences in microbial community composition between groups. Standard implementations of these methods available in PAST were used and did not explicitly account for the paired structure of the repeated measurements. No formal correction for multiple comparisons was applied because this was an exploratory pilot study intended to identify potential microbiota changes associated with the intervention. Statistical significance was established at *p* < 0.05.

To identify taxa contributing most substantially to intergroup differences, SIMPER analysis [46] was applied. In addition, Uniform Manifold Approximation and Projection (UMAP) analysis based on the Bray–Curtis distance matrix was used to visualize similarities and differences among microbiome samples [47]. All statistical analyses were performed using PAST version 4.17 software [48].

## 3. Results

### Diversity of Microbiota Composition

The overall assessment of alpha diversity within the studied cohort, both in the entire sample and after stratification into the control group (C, healthy individuals), the obesity group before initiation of the NW +TRE program (A), and the same group following completion of the intervention (B), demonstrated an increase in species diversity with decreasing taxonomic rank. The highest diversity values were observed at the species level, whereas the lowest values were recorded at the phylum level (Table 3).

Notably, lower alpha-diversity values (Simpson 1-D and Shannon H indices) were observed in the control group compared with group A prior to initiation of the NW + TRE program at the phylum, class, and order taxonomic levels. However, this trend was reversed at the genus and species levels, where higher diversity indices were observed in the control group and lower values in group A.

A further important observation was a trend toward increased alpha diversity in the intervention group following completion of the NW + TRE program (group B) compared with baseline values (group A) (Table 3).

Interestingly, the diversity index values observed in the intervention group following completion of the NW + TRE program (group B) approached those recorded in the control group (C) at the species, genus, and family taxonomic levels. Higher estimated index values indicate greater microbiota diversity and a more even distribution of microbial taxa.

Statistically significant differences were identified between group A (before the intervention) and the control group (C) at the order, family, class, and phylum taxonomic levels (Table 4), as well as between group B and the control group (C) at the order level. These findings may suggest that the 6-week NW + TRE intervention contributed to remodeling of microbiota diversity and to a reduction in overall microbial dissimilarity in group B relative to the control group.

Statistically significant differences were observed between the study group before the intervention (A) and the control group (C) in the overall mean dissimilarity across all taxonomic levels, which may indicate substantial differences in microbiota composition between the two groups. The observed reduction in the statistical significance of differences between the post-intervention group (B) and the control group across successive taxonomic levels may tentatively indicate a trend toward lower microbiota compositional dissimilarity between these groups; however, this observation should not be considered direct biological evidence of such a change.

However, it should be emphasized that changes in the level of statistical significance (*p*-value) do not constitute a direct measure of biological similarity, as *p*-values are also influenced by data variability and sample size. Therefore, these findings should be interpreted together with the observed reduction in differences between the mean values of the analyzed diversity measures (Table 5). Within this context, the results may suggest that the physical activity program (NW) combined with TRE may have contributed to a partial reorganization of the gut microbiota in individuals with obesity toward a profile more closely resembling that observed in individuals with normal body weight.

Overall average dissimilarity calculated between the A and C groups using the Bray–Curtis measure showed significant differences at every taxonomic level. Following completion of the 6-week NW + TRE program, the overall mean microbiota dissimilarity relative to the control group remained statistically significant (B/C). Only at the phylum level, no statistically significant differences were observed. The higher *p*-values observed for the B/C comparisons compared with the A/C comparisons may indicate a potential trend toward decreasing differences in the microbiome diversity measure. Furthermore, no significant differences in mean dissimilarity were detected between the microbiota of individuals with obesity before and after the NW intervention (A/B; Table 5).

Analysis of relative abundance identified several taxa that were characteristic of specific study groups (Supplementary Table S2). At the phylum level, *Firmicutes* was identified as characteristic of the control group and, following the intervention, also of group B, whereas *Proteobacteria* was characteristic of the control group only. At the class level, *Clostridia* was characteristic of both the control group and group B, while *Gammaproteobacteria*, *Negativicutes*, and *Bacilli* were characteristic of the control group. In contrast, *Coriobacteriia* was characteristic of group B. At the family level, *Lachnospiraceae* was characteristic of the control group and group B, whereas *Oscillospiraceae*, *Streptococcaceae*, *Eubacteriaceae*, *Veillonellaceae*, and *Enterobacteriaceae* were characteristic of the control group. *Clostridiaceae* was identified as characteristic of group B. At the order level, *Eubacteriales* was characteristic of the control group and group B, whereas *Lactobacillales*, *Enterobacterales*, and *Veillonellales* were characteristic of the control group.

At the genus level, *Blautia*, *Bifidobacterium*, *Fusicatenibacter*, *Mediterraneibacter*, *Anaerobutyricum*, *Anaerostipes*, *Klebsiella*, *Dorea*, *Streptococcus*, *Ruminococcus*, and *Eubacterium* were characteristic of the control group, whereas *Collinsella* was characteristic of group B. At the species level, *Fusicatenibacter saccharivorans* and *Klebsiella pneumoniae* were characteristic of the control group, while *Clostridium saudiense* was characteristic of group B. Complete relative abundance data are presented in Supplementary Table S2. Across multiple taxonomic levels, a rearrangement of the microbiota toward a profile characteristic of the control group was observed. This included, among others, the classes *Clostridia, Bacilli*, and *Bacteroidia*; the families *Bifidobacteriaceae*, *Coprobacillaceae*, and *Clostridiaceae*; the orders *Eubacteriales* and *Lactobacillales*; as well as individual species such as *Blautia luti* and *Eubacterium rectale*. In the obesity group, the genus *Holdemanella* was identified as a characteristic taxon. Furthermore, analysis of mean dissimilarity identified key taxa contributing to intergroup differentiation, including *Firmicutes*, *Blautia*, *Clostridia*, *Lachnospiraceae*, *Eubacteriales*, and *Bifidobacterium longum* (Supplementary Figures S1–S5).

## 4. Discussion

A review of the literature regarding the microbiota composition of individuals with obesity indicates increased abundance of taxa such as *Firmicutes*, *Proteobacteria*, *Fusobacteria*, an elevated *Firmicutes/Bacteroidetes* ratio, and *Lactobacillus* spp. [28,49]. A similar pattern was also observed in our cohort of adults with obesity. Importantly, participation in the 6-week NW and TRE intervention was associated with a reduction in the *Firmicutes/Bacteroidetes* (F/B) ratio, reaching a value even lower than that observed in the control group (control group: 59.35; obesity group before the 6-week NW + TRE intervention: 72.1; obesity group after the intervention: 47.12).

The microbiota of individuals with obesity is also characterized by lower abundance of taxa such as *Bacteroidetes*, *Faecalibacterium prausnitzii*, *Akkermansia muciniphila*, *Methanobrevibacter smithii*, and *Bifidobacterium animalis* [29]. Our findings are consistent with these observations. Prior to the NW + TRE intervention, the abundance of the aforementioned taxa was reduced, whereas numerical increases were observed for several taxa observed following completion of the program. Exceptions included *A. muciniphila* and *F. prausnitzii*, for which no substantial changes were detected.

A possible functional interpretation of the observed microbial shifts suggests that the reduction in the *Firmicutes/Bacteroidetes* ratio and the partial recovery of taxa such as *Bifidobacterium* and *Faecalibacterium* may reflect a transition toward a more metabolically favorable gut ecosystem. These taxa are probably associated with enhanced production of short-chain fatty acids, improved intestinal barrier integrity, and modulation of low-grade systemic inflammation, all of which are relevant in the context of obesity-related metabolic dysfunction. Although no direct functional metagenomic or metabolomic measurements were performed in the present study, the observed taxonomic trends may indirectly indicate a shift toward increased microbial metabolic capacity supporting host energy homeostasis. At the same time, the absence of consistent changes in *Akkermansia muciniphila* and *Faecalibacterium prausnitzii* highlights the heterogeneity of individual responses to dietary and time-restricted interventions and suggests that microbial restructuring may occur in a taxon-specific and temporally delayed manner. Future studies integrating metagenomic and metabolomic approaches will be necessary to determine whether these compositional changes translate into measurable functional outcomes and to better disentangle causality from association in microbiota–host interactions.

Small sample sizes are a common limitation in exploratory microbiome intervention studies and primarily reduce the statistical power to detect subtle changes in low-abundance taxa, particularly in longitudinal within-subject analyses. However, repeated measurements obtained from the same individual substantially reduce inter-individual variability and increase the sensitivity of detecting intervention-related microbial shifts [50]. Importantly, recent evidence highlights that microorganisms belonging to the rare biosphere may play a disproportionately important ecological role in shaping community dynamics and functional transitions [51,52]. Although these taxa often do not reach statistical significance after correction for multiple testing in cross-sectional analyses, their emergence or decline may represent early indicators of microbiome restructuring and a shift between homeostatic and dysbiotic states [52]. Therefore, in exploratory longitudinal studies such as ours, observed numerical changes in low-abundance taxa should not be interpreted exclusively through the lens of statistical significance, but rather considered in the broader context of ecological relevance, temporal dynamics, and potential functional impact of the microbiome [53].

Unfortunately, it is still relatively common to encounter articles in ecological journals in which statistical significance is confused with biological relevance or with the strength of evidence against the null hypothesis. While statistical analysis is essential, the biological role of organisms should not be disregarded solely because they are of low abundance, were previously undetected, or appear only transiently in a given dataset [54].

Researchers explicitly emphasize that statistical significance is neither a necessary nor a sufficient condition for biological relevance. In many cases, statistical outcomes may be influenced by sequencing depth or sample size rather than reflecting true biological effects. Instead, authors advocate focusing on effect size and confidence intervals, which more accurately capture the actual impact of microbiota compositional changes on the host organism [53,54,55,56].

The use of the primer pair 337F and 1391R enables amplification of a long fragment of the 16S rRNA gene, encompassing regions V3 to V9. This approach is particularly appropriate for long-read sequencing technologies (e.g., Oxford Nanopore Technologies), where full-length amplicons are sequenced. Historically, PCR-based methods have expanded the application not only of the 16S rRNA gene itself but also of the intergenic spacer region between 16S and 23S rRNA genes for bacterial taxonomy [25,57].

The V3–V9 region covers seven of the nine hypervariable regions of the 16S rRNA gene, providing substantially greater phylogenetic resolution compared to standard short amplicons (e.g., V3–V4). The widely used V3–V4 fragment represents a short single amplicon of approximately 400–460 bp [11,12]. Given the inclusion of a higher number of variable regions, this approach provides considerably more genetic information, which directly translates into improved sensitivity for detecting rare taxa and more precise taxonomic assignment, ultimately enhancing phylogenetic resolution [58,59,60].

In turn, the application of nanopore sequencing technology (Oxford Nanopore Technologies—ONT, Oxford, United Kingdom) to V3–V9 amplicons (~1050 bp) significantly alters the comparability with historical datasets. While it enables a new generation of analytical algorithms, it also introduces specific technical and bioinformatic challenges [61].

Future research should extend beyond taxonomic profiling by incorporating metagenomic and metabolomic analyses to identify functional shifts in microbial pathways, and should further disentangle the individual and synergistic effects of physical activity and time-restricted eating using randomized, factorial study designs with longer intervention and washout periods.

### Study Limitations

This study has several limitations that should be considered when interpreting the findings. First, the small sample size (*n* = 10 per group) limited the statistical power to detect small- and moderate-sized effects, particularly in the alpha-diversity analysis and the pre–post intervention comparison. A formal a priori power analysis was not feasible due to the lack of previous data on the expected effects of a combined NW and TRE intervention on the gut microbiota. Therefore, the present findings should be considered hypothesis-generating and require confirmation in larger studies with adequate statistical power.

Second, environmental conditions during the outdoor training sessions, including ambient temperature, humidity, and wind speed, were not monitored and may have contributed to variability in the physiological response to exercise.

Third, participants’ diet was not standardized. Although this represents a limitation, it also constitutes a strength of the study, as the intervention intentionally focused solely on restricting the eating window (TRE) without modifying diet composition or caloric intake. This approach, in our opinion, better reflects the real-world implementation of TRE and enhances the practical applicability of the findings.

No formal correction for multiple comparisons was applied because of the exploratory nature of this pilot study. Consequently, statistically significant findings, particularly those obtained from numerous taxonomic comparisons, should be interpreted with caution and regarded as hypothesis-generating. Future studies with larger cohorts and appropriate adjustment for multiple testing are needed to confirm these observations. Furthermore, although the paired structure of the pre–post comparisons was appropriately accounted for in the alpha-diversity analyses, the beta-diversity analyses were performed using the standard ANOSIM and PERMANOVA procedures available in PAST, which do not explicitly account for repeated measurements. Therefore, the longitudinal beta-diversity findings should also be interpreted with appropriate caution.

## 5. Conclusions

The NW and TRE intervention implemented in individuals with obesity contributed to increased gut microbiota diversity. The elevated *Firmicutes/Bacteroidetes* ratio, considered by some authors to be characteristic of obesity, was reduced following the intervention. Simultaneously, an increased abundance of symbiotic bacterial taxa, including *Akkermansia*, *Faecalibacterium*, *Eubacterium*, *Anaerostipes*, *Blautia*, and *Coprococcus*, was observed.

The obtained findings suggest that a 6-week NW program may support obesity treatment through modulation of gut microbiota composition toward a healthier symbiotic profile, accompanied by reductions in body weight, BMI, and adipose tissue mass.

## Figures and Tables

**Table 1:** Inclusion and exclusion criteria for participant recruitment, including medical contraindications to Nordic Walking (NW) and time-restricted eating (TRE) interventions.

Inclusion Criteria	Exclusion Criteria
Age ≥ 18 years Written informed consent BMI < 30.0 kg/m^2^ (obesity group) BMI 18.5–24.9 kg/m^2^ (control group) No contraindications to structured physical activity No contraindications to time-restricted eating (TRE) Willingness to participate in 6-week Nordic Walking (NW) program Willingness to adhere to TRE protocol Ability to safely perform moderate-intensity exercise Stable lifestyle (diet and physical activity) for ≥3 months prior to enrollment (recommended)	Chronic or uncontrolled endocrine disorders (e.g., uncontrolled diabetes mellitus, thyroid dysfunction) Active infection at baseline or need for antibiotic therapy Antibiotic therapy within 3 months prior to enrollment Probiotic or prebiotic supplementation within 3 months prior to enrollment Use of anti-inflammatory or immunomodulatory medications at baseline Gastrointestinal diseases affecting digestion or absorption (e.g., inflammatory bowel disease, Crohn’s disease, ulcerative colitis, celiac disease) History of malignancy within the past 5 years (less than 5 years after cancer remission) History of eating disorders (e.g., anorexia nervosa, bulimia nervosa)—contraindication to TRE Type 1 diabetes mellitus or insulin-dependent diabetes (risk of hypoglycemia during fasting windows) Severe cardiovascular disease- contraindication to NW Neurological or musculoskeletal disorders limiting safe gait or weight-bearing exercise (e.g., severe osteoarthritis, advanced neuropathy, recent fractures)—contraindication to NW Pregnancy or lactation Significant weight change (>5% body mass) within 3 months prior to enrollment (recommended)

**Table 2:** Participant characteristics (*n* = 20).

		Median	Mean ± DS	Min	Max	*p t*-Test	Cohen’s d
BH [cm]	A	159.80	161.28 ± 5.075	155.5	170.0	0.060	−0.927
	C	167.00	167.05 ± 7.100	156.0	178.0		
BM [kg]	A	78.80	80.99 ± 8.300	70.20	94.50	0.019	1.235
	C	71.00	69.22 ± 10.620	51.70	80.90		
LBM [kg]	A	47.40	49.28 ± 5.150	43.20	57.30	0.617	0.234
	C	45.65	47.39 ± 9.965	34.10	65.00		
SLM [kg]	A	42.90	44.73 ± 4.695	39.30	52.20	0.365	0.428
	C	39.30	40.93 ± 11.386	26.60	59.10		
TBW [%]	A	44.30	43.79 ± 1.265	41.60	45.20	<0.001	−2.072
	C	46.90	47.20 ± 1.923	44.80	50.60		
BMI	A	31.50	31.83 ± 1.734	30.30	34.30	<0.001	2.892
	C	24.69	24.97 ± 2.696	21.05	29.00		
Body fat [%]	A	38.50	39.14 ± 1.806	37.20	42.30	0.003	1.559
	C	34.00	33.82 ± 4.374	27.20	40.40		

Abbreviations: A—intervention group; C—control group; BH, body height; BM, body mass; LBM, lean body mass; SLM, soft lean mass; TBW, total body water; BMI, body mass index; Body fat [%], percentage of body fat. Units are given in square brackets.

**Table 3:** Studied alpha-diversity metrics: Shannon Index and Simpson’s Index (1-D) parameter (median) considering the taxonomic levels of the microbiota and the division of the study sample into groups.

Taxon	Group	Shannon H	Lower 95%	Upper 95%	Simpson 1-D	Lower 95%	Upper 95%
Phyllum	Control	0.61	0.61	0.61	0.23	0.20	0.23
	Group A	0.69	0.68	0.69	0.25	0.25	0.25
	Group B	0.61	0.60	0.61	0.23	0.23	0.23
	All	0.65	0.65	0.65	0.24	0.24	0.24
Class	Control	1.08	1.08	1.08	0.20	0.20	0.20
	Group A	1.16	1.16	1.16	0.21	0.21	0.21
	Group B	1.09	1.08	1.09	0.20	0.20	0.20
	All	1.15	1.15	1.15	0.21	0.21	0.21
Family	Control	1.66	1.66	1.67	0.10	0.10	0.10
	Group A	1.78	1.77	1.78	0.11	0.11	0.12
	Group B	1.82	1.81	1.82	0.12	0.12	0.12
	All	1.80	1.80	1.80	0.12	0.12	0.12
Order	Control	1.11	1.10	1.11	0.14	0.14	0.14
	Group A	1.22	1.21	1.22	0.15	0.15	0.15
	Group B	1.15	1.15	1.16	0.14	0.14	0.14
	All	1.20	1.20	1.20	0.15	0.15	0.15
Genus	Control	3.16	3.16	3.17	0.21	0.21	0.21
	Group A	2.92	2.91	2.92	0.16	0.16	0.16
	Group B	3.09	3.09	3.10	0.19	0.19	0.19
	All	3.20	3.20	3.20	0.21	0.21	0.22
Species	Control	3.73	3.72	3.73	0.30	0.30	0.30
	Group A	3.64	3.63	3.64	0.27	0.27	0.27
	Group B	3.69	3.68	3.69	0.29	0.28	0.29
	All	3.84	3.84	3.84	0.33	0.33	0.33

A—group of participants before starting the NW + TRE program; B—group of participants after completing the Nordic walking program.

**Table 4:** Assessment of the significance of differences between the studied groups.

Alpha Diversity			
Index	A-B ^#^	A-C ^##^	B-C ^##^
		Pairwise (*p*)	
	Species		
Shannon_H	0.57	0.78	0.97
Simpson 1-D	0.57	0.84	0.97
	Genus		
Shannon_H	0.62	0.39	0.54
Simpson 1-D	0.57	0.35	0.78
	Order		
Shannon_H	0.62	0.01 *	0.08 *
Simpson 1-D	0.57	0.01 *	0.05 *
	Family		
Shannon_H	0.73	0.21	0.10
Simpson 1-D	0.97	0.02 *	0.07 *
	Class		
Shannon_H	0.62	0.02 *	0.13
Simpson 1-D	0.24	0.02 *	0.11
	Phyllum		
Shannon_H	0.62	0.02 *	0.31
Simpson 1-D	0.52	0.39	0.97

A—group of patients before starting the NW + TRE program; B—group of patients after completing the program; C—control group; *—statistically significant *p*-value; #—Wilcoxon’s signed rank test; ##—Mann–Whitney U test.

**Table 5:** Overall average dissimilarity calculated between the study groups using the Bray–Curtis measure.

Taxon	Phyllum	Class	Family	Order	Genus	Species
A/Control	49.98	52.57	56.05	52.72	60.99	65.97
*p*	0.02 *	0.005 *	0.002 *	0.0048 *	0.0006 *	0.0006 *
A/B	25.24	31.2	38.22	44.38	46.93	51.86
*p*	1	1	0.882	1	1	1
B/Control	39.46	44.1	50.48	31.62	58.09	63.47
*p*	0.14	0.015 *	0.011 *	0.022 *	0.005 *	0.0003 *

A—group of patients before starting the NW program; B—group of patients after completing the Nordic walking program; control group for all levels. * Statistically significant value.

## Data Availability

The data is available from the corresponding author on request.

## Supplementary Materials

The following supporting information can be downloaded at: https://www.mdpi.com/article/10.3390/nu18142373/s1.
