# Supplementary material for: Nordic Walking Combined with Time-Restricted Eating Is Associated with Changes in Gut Microbiota Composition in Adults with Obesity—Pilot Study
Source: Nutrients. 2026 Jul 20;18(14):2373. doi: 10.3390/nu18142373 (PMC13414784; doi:10.3390/nu18142373)
Supplement: Supplementary file 1 [file nutrients-18-02373-s001.zip › nutrients-4379161-supplementary.pdf]

Table S1 Overview of Methods, Sequencing Technologies, Molecular Markers, and Reference Databases Used for Taxonomic Identification and Microbiota Analysis

Methods and Sequencing Technologies		
Approach / Technology	Description & Characteristics	References
Culture-Based Methods	Traditional approach for investigating microbiota composition.	[1]
Genetic Analyses	Modern approach analyzing the genetic material of microorganisms across various environments.	[2,3]
Next-Generation Sequencing (NGS)	Modern, rapid, and cost-effective sequencing technologies used for genetic analysis.	[4]
The "Gold Standard" 16S rRNA Gene		
Gene Property / Feature	Details & Specifications	References
Role	Encodes the small ribosomal subunit; present in all bacteria. Widely used for bacterial identification.	[4,5]
Conserved Regions	Identical across bacterial taxa; facilitates technical assay performance.	[4,5]
Variable Regions	Regions V1 through V9 which are unique to specific taxonomic groups.	[6,7]
Resolution	Enables identification at the genus level, and usually to the species level.	[4]
Fragment Length	Approximately 1,500 base pairs.	[4]
Comparative Microbiological Databases		
Comparative Microbiological Databases	Function & Specialization	References
SILVA	A widely used comparative database for rRNA data.	[8]
Greengenes	A extensively utilized database providing annotated and aligned 16S rRNA sequences.	[9]
GenBank	Primary sequence database maintained by NCBI; features the BLAST comparative tool.	[10,11]
ENA	European Nucleotide Archive; maintained by EMBL-EBI and synchronized globally.	[12]
Ribosomal Database Project	Specialized in rRNA sequences; provides high-quality datasets and microbiota classification tools.	[13]
LPSN	List of Prokaryotic names with Standing in Nomenclature; contains official bacterial nomenclature.	[14]
Other genes enabling taxonomic identification		
		References
rpoB<br>gyrB<br>recA	RNA polymerase β-subunit DNA gyrase subunit B recombination protein A	[15-20]
16S and 23S rRNA genes	intergenic spacer regions (ITS), which are DNA fragments located between rRNA-coding genes	[15-20]

Table S2 Relative abundance calculated within the group at different taxonomic levels (relative abundance >0,01 at least in one of the groups).

Relative Abundance			Phyllum
Control (C)	A	B	
0.831*	0.721	0.801**	*Firmicutes*

0.094	0.259	0.171	*Actinobacteria*
0.060*	0.007	0.007	*Proteobacteria*
0.014	0.010	0.017	*Bacteroidetes*
Relative Abundance			Class
Control	A	B	
0.727*	0.655	0.703**	*Clostridia*
0.059*	0.006	0.006	*Gammaproteobacteria*
0.080	0.133	0.074	*Actinomycetia*
0.037*	0.004	0.001	*Negativicutes*
0.049*	0.024	0.035	*Bacilli*
0.015	0.105	0.114*	*Coriobacteriia*
0.014	0.010	0.017	*Bacteroidia*
0.017	0.059	0.044	*Erysipelotrichia*
Relative Abundance			Family
Control	A	B	
0.602*	0.518	0.543**	*Lachnospiraceae*
0.081	0.137	0.076	*Bifidobacteriaceae*
0.065*	0.083	0.071	*Oscillospiraceae*
0.015	0.042	0.039	*Erysipelotrichaceae*
0.042*	0.020	0.026	*Streptococcaceae*
0.002	0.019	0.006	*Coprobacillaceae*
0.013	0.017	0.033	*Peptostreptococcaceae*
0.007	0.016	0.023	*Eggerthellaceae*
0.020	0.016	0.030**	*Clostridiaceae*
0.022*	0.007	0.009	*Eubacteriaceae*
0.006	0.005	0.014	*Bacteroidaceae*
0.038*	0.003	0.001	*Veillonellaceae*
0.055*	0.003	0.002**	*Enterobacteriaceae*
Relative Abundance			Order
Control	A	B	
0.729*	0.657	0.705**	*Eubacteriales*
0.008	0.090	0.093	*Coriobacteriales*
0.078	0.131	0.072	*Bifidobacteriales*
0.017	0.059	0.044	*Erysipelotrichales*
0.047*	0.023	0.033	*Lactobacillales*
0.007	0.015	0.022	*Eggerthellales*
0.014	0.010	0.017	*Bacteroidales*
0.056*	0.003	0.003	*Enterobacterales*
0.037*	0.003	0.001	*Veillonellales*
Relative Abundance			Genus
Control	A	B	

0.193*	0.286	0.255	*Blautia*
0.087*	0.138	0.080	*Bifidobacterium*
0.078*	0.018	0.017	*Fusicatenibacter*
0.074*	0.025	0.024	*Mediterraneibacter*
0.061*	0.053	0.061	*Anaerobutyricum*
0.054*	0.031	0.040	*Anaerostipes*
0.051*	0.000	0.000	*Klebsiella*
0.048*	0.042	0.044	*Dorea*
0.043*	0.019	0.026	*Streptococcus*
0.040	0.003	0.001	*Dialister*
0.029*	0.032	0.017	*Ruminococcus*
0.024*	0.007	0.009	*Eubacterium*
0.018	0.031	0.046	*Gemmiger*
0.017	0.013	0.028	*Clostridium*
0.016	0.017	0.024	*Coprococcus*
0.013	0.024	0.032	*Faecalibacterium*
0.008	0.012	0.024	*Romboutsia*
0.008	0.086	0.094**	*Collinsella*
0.006	0.013	0.008	*Longibaculum*
0.002	0.019	0.006	*Catenibacterium*
0.000	0.016	0.022	*Holdemanella*
0.006	0.004	0.013	*Bacteroides*

Relative Abundance			Species
Control	A	B	
0.053	0.117	0.092	*Blautia luti*
0.008	0.085	0.088	*Collinsella aerofaciens*
0.063	0.018	0.074	*Eubacterium rectale*
0.062	0.056	0.062	*Anaerobutyricum hallii*
0.024	0.053	0.053	*Blautia obeum*
0.019	0.032	0.047	*Gemmiger formicilis*
0.026	0.061	0.045	*Blautia wexlerae*
0.054	0.032	0.040	*Anaerostipes hadrus*
0.013	0.025	0.032	*Faecalibacterium prausnitzii*
0.038	0.031	0.031	*Dorea longicatena*
0.076	0.047	0.025	*Bifidobacterium longum*
0.041	0.024	0.025	*Blautia faecis*
0.000	0.017	0.022	*Holdemanella biformis*
0.007	0.012	0.021	*Romboutsia timonensis*
0.011	0.014	0.021	*Coprococcus comes*
0.003	0.050	0.021	*Bifidobacterium adolescentis*
0.007	0.008	0.019**	*Clostridium saudiense*
0.056	0.020	0.018	*Mediterraneibacter faecis*
0.080*	0.019	0.017	*Fusicatenibacter saccharivorans*

0.025	0.014	0.015	*Blautia stercoris*
0.025	0.030	0.015	*Ruminococcus bromii*
0.011	0.013	0.013	*Dorea formicigenerans*
0.006	0.014	0.010	*Streptococcus thermophilus*
0.006	0.014	0.009	*Longibaculum muris*
0.003	0.020	0.008	*Bifidobacterium faecale*
0.013	0.001	0.007	*Streptococcus salivarius*
0.002	0.020	0.006	*Catenibacterium mitsuokai*
0.016	0.004	0.005	*Eubacterium coprostanoligenes*
0.041	0.003	0.001	*Dialister invisus*
0.024	0.000	0.000	*Klebsiella variicola*
0.020*	0.000	0.000	*Klebsiella pneumoniae*

* - Group-specific species in comparative analysis of groups: Control, A, B;

** - Group-specific species in comparative analysis of groups: A, B;

Indicator species analysis, p<=0,05.

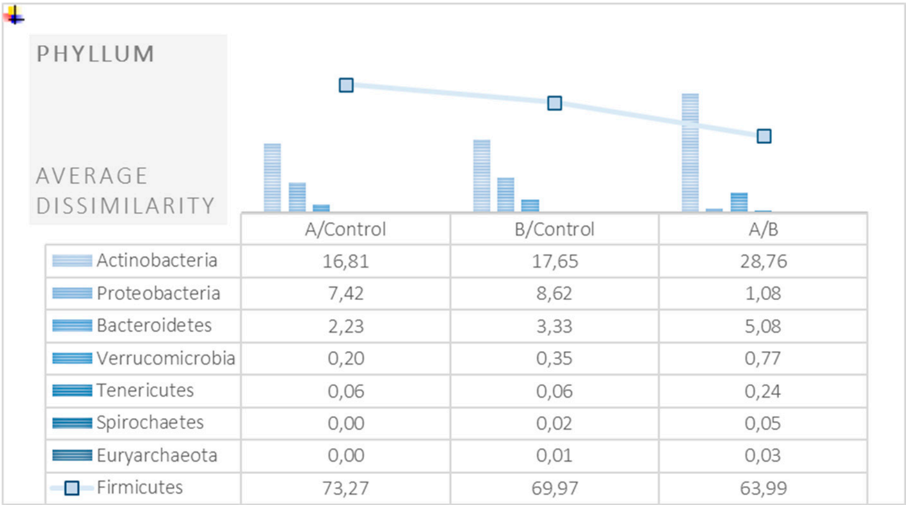

Figure S1 Mean divergence between the study groups (A-group of patients before starting the NW +TRE program; B-group of patients, Control) after completing the program at the phylum level.

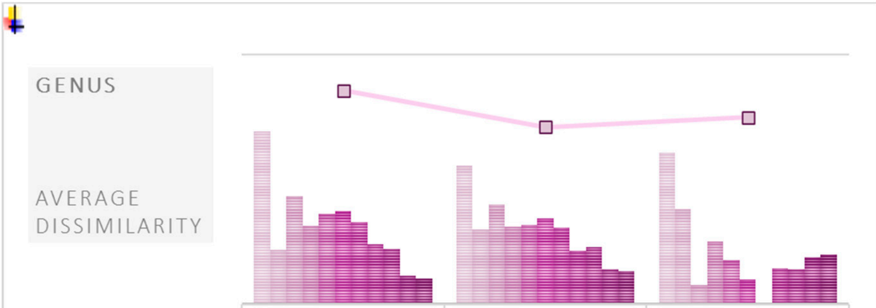

	A/Control	B/Control	A/B
Bifidobacterium	6,95	5,56	6,09
Collinsella	2,20	3,01	3,82
Fusicatenibacter	4,36	4,00	0,76
A0erobutyricum	3,15	3,11	2,52
A0erostipes	3,62	3,17	1,79
Mediterraneibacter	3,75	3,44	0,99
Klebsiella	3,30	3,08	0,00
Dorea	2,40	2,14	1,46
Streptococcus	2,22	2,31	1,42
Faecalibacterium	1,15	1,40	1,87
Gemmiger	1,03	1,35	2,02
Blautia	8,58	7,11	7,51

Figure S2 Mean divergence between the study groups (A-group of patients before starting the NW + TRE program; B-group of patients, Control) after completing the program at the genus level.

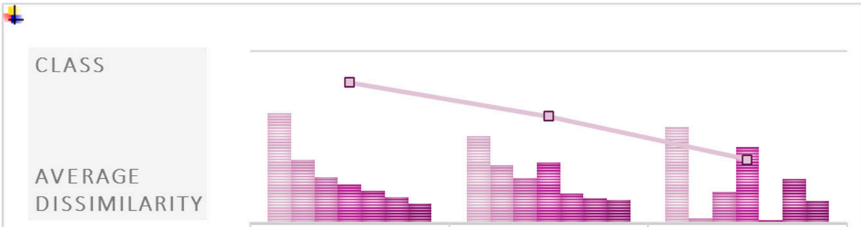

	A/Control	B/Control	A/B
Actinomycetia	6,40	5,05	5,58
Gammaproteobacteria	3,68	3,36	0,26
Bacilli	2,64	2,60	1,83
Coriobacteriia	2,22	3,52	4,40
Negativicutes	1,84	1,68	0,17
Erysipelotrichia	1,52	1,41	2,58
Bacteroidia	1,12	1,31	1,28
Clostridia	32,68	24,88	14,74

Figure S3 Mean divergence between the study groups (A-group of patients before starting the NW + TRE program; B-group of patients, Control) after completing the program at the class level.

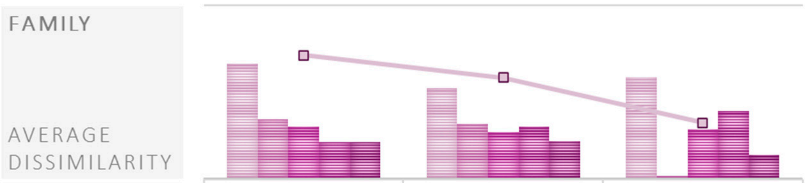

	A/Control	B/Control	A/B
Bifidobacteriaceae	6,64	5,26	5,86
Enterobacteriaceae	3,48	3,21	0,22
Oscillospiraceae	3,06	2,72	2,88
Coriobacteriaceae	2,17	3,03	3,91
Streptococcaceae	2,17	2,21	1,40
Lachnospiraceae	28,62	23,46	13,08

Figure S4 Mean divergence between the study groups (A-group of patients before starting the NW + TRE program; B-group of patients, Control) after completing the program at the family level.

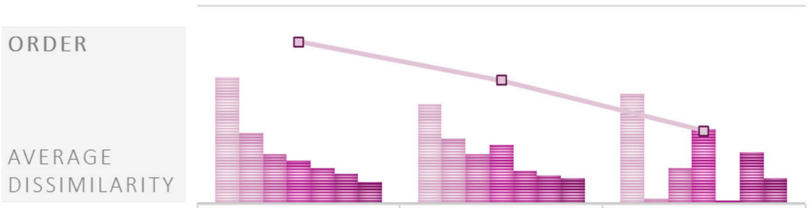

	A/Control	B/Control	A/B
Bifidobacteriales	6,39	5,03	5,57
Enterobacterales	3,59	3,30	0,25
Lactobacillales	2,52	2,53	1,81
Coriobacteriales	2,18	3,01	3,79
Veillonellales	1,81	1,65	0,16
Erysipelotrichales	1,52	1,42	2,59
Bacteroidales	1,12	1,31	1,28
Eubacteriales	32,74	24,96	14,78

Figure S5 Mean divergence between the study groups (A-group of patients before starting the NW + TRE program; B-group of patients, Control) after completing the program at the order level.

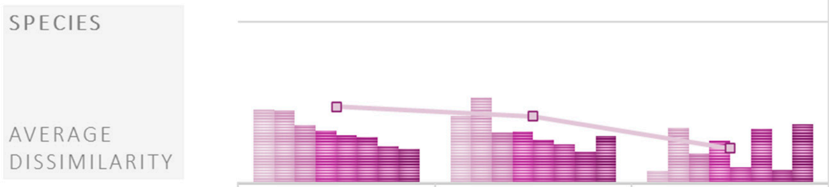

	A/Control	B/Control	A/B
Fusicatenibacter saccharivorans	4,547	4,139	0,7686
Eubacterium rectale	4,508	5,313	3,414
Anaerostipes hadrus	3,62	3,125	1,858
Anaerobutyricum hallii	3,264	3,2	2,612
Mediterraneibacter faecis	2,946	2,71	0,9793
Blautia luti	2,874	2,434	3,399
Blautia faecis	2,276	1,942	0,8041
Collinsella aerofaciens	2,111	2,892	3,638
Bifidobacterium longum	4,735	4,15	2,157

Figure S6 Mean divergence between the study groups (A-group of patients before starting the NW + TRE program; B-group of patients, Control) after completing the program at the species level.

References

1. Gajic, I.; Jovicevic, M.; Kekic, D.; Kabic, J.; Vicic, I.; Lukovic, B.; Tomic, A.; Sovljanski, O.; Skoric, M.; Sikanic, I.; et al. Evolving Approaches to Bacterial Identification: A Review of Classical and Modern Techniques. Int. J. Mol. Sci. **2026**, 27, 5092. https://doi.org/10.3390/ijms27115092
2. Ursell, L.K.; Metcalf, J.L.; Parfrey, L.W.; Knight, R. Defining the human microbiome. Nutr Rev. **2012**, 70 Suppl 1. S38-44. https://doi.org/10.1111/j.1753-4887.2012.00493.x
3. Fraher, M.H.; O’Toole, P.W.; Quigley, E.M.M. Techniques Used to Characterize the Gut Microbiota: A Guide for the Clinician. Nat. Rev. Gastroenterol. Hepatol. **2012**, 9, 312–322. https://doi.org/10.1038/nrgastro.2012.44
4. Campodónico, V.L.; Ruelle, J.; Fitzgerald, A.; Bergman, Y.; Osborne, B.; Bourdas, D.; Lu, J.; Carroll, K.C.; Simner, P.J. Evaluation of long-read 16S rRNA next-generation sequencing for identification of bacterial isolates in a clinical diagnostic laboratory. J Clin Microbiol **2025**, 63, e01670-24. https://doi.org/10.1128/jcm.01670-24
5. Woese, C.R.; Fox, G.E. Phylogenetic structure of the prokaryotic domain: the primary kingdoms. Proc Natl Acad Sci U S A. **1977**, 74, 5088-90. https://doi.org/10.1073/pnas.74.11.5088
6. Vargas-Albores, F.; Ortiz-Suárez, L.E.; Villalpando-Canchola, E.; Martínez-Porchas, M. Size-Variable Zone in V3 Region of 16S rRNA. RNA Biol. **2017**, 14, 1514–1521. https://doi.org/10.1080/15476286.2017.1317912
7. Thijs, S.; Op De Beeck, M.; Beckers, B.; Truyens, S.; Stevens, V.; Van Hamme, J.D.; Weyens, N.; Vangronsveld, J. Comparative Evaluation of Four Bacteria-Specific Primer Pairs for 16S rRNA Gene Surveys. Front. Microbiol. **2017**, 8, 494. https://doi.org/10.3389/fmicb.2017.00494
8. SILVA Team. SILVA Ribosomal RNA Gene Database. Available online: https://www.arb-silva.de/ (accessed on 19 March 2026).
9. McDonald, D. Introducing Greengenes2 2022.10. QIIME 2 Forum. Available online: https://forum.qiime2.org/t/introducing-greengenes2-2022-10/25291 (accessed on 19 March 2026).
10. Altschul SF, Gish W, Miller W, Myers EW, Lipman DJ. Basic local alignment search tool. J Mol Biol. **1990**;215(3):403-410. https://doi.org/10.1016/S0022-2836(05)80360-2
11. National Center for Biotechnology Information (NCBI). BLAST: Basic Local Alignment Search Tool. Available at: https://blast.ncbi.nlm.nih.gov/Blast.cgi (accessed 19 March 2026).
12. European Molecular Biology Laboratory–European Bioinformatics Institute (EMBL-EBI). European Nucleotide Archive (ENA). https://www.ebi.ac.uk/ena/browser/home. Accessed 19 March 2026.
13. Ribosomal Database Project. Michigan State University. https://rdp.cme.msu.edu/; accessed 19 March 2026.
14. Leibniz Institute DSMZ – German Collection of Microorganisms and Cell Cultures. List of Prokaryotic names with Standing in Nomenclature (LPSN). https://lpsn.dsmz.de/; accessed 19 March 2026.
15. Case, R. J.; Boucher, Y.; Dahllöf, I.; Holmström, C.; Crosby, W. J.; & Kjelleberg, S. Use of 16S rRNA and rpoB genes as molecular markers for microbial community analysis. Appl. Environ. Microbiol. **2007**, 73, 278-288. https://doi.org/10.1128/AEM.01177-06
16. Yamamoto, S.; Harayama, S. PCR amplification and direct sequencing of gyrB genes with universal primers and their application to the detection and phylogenetic analysis of Pseudomonas strains. Appl. Environ. Microbiol. **1995**, 61, 1104-1109. https://doi.org/10.1128/aem.61.3.1104-1109.1995
17. Mahenthiralingam, E.; Bischof, J.; Byrne, S.K.; McConville, C.; Kelly, B.; Speert, D.P. DNA sequence analysis of a highly polymorphic DNA region bordered by the recA and the orfP genes of Burkholderia cepacia complex. J. Clin. Microbiol. **2000**, 38, 3165-3173. https://doi.org/10.1128/jcm.38.9.3165-3173.2000.
18. Gürtler, V.; Stanisich, V.A. New approaches to typing and identification of bacteria using the 16S-23S rDNA spacer region. Microbiology, **1996**, 142, 3-16. https://doi.org/10.1099/13500872-142-1-3
19. Nowak, A.; Kur, J. Genomic species typing of Acinetobacters by polymerase chain reaction amplification of the recA gene. FEMS Microbiol. Lett. **1995**, 130, 327–332. https://doi.org/10.1111/j.1574-6968.1995.tb07739.x

20. Nowak, A.; Burkiewicz, A.; Kur, J. PCR differentiation of seventeen genospecies of Acinetobacter. FEMS Microbiol. Lett. **1995**, 126, 181–187. https://doi.org/10.1111/j.1574-6968.1995.tb07414.x
